# Prognostic and staging implications of mandibular canal invasion in lower gingival squamous cell carcinoma

**DOI:** 10.1002/cam4.899

**Published:** 2016-10-19

**Authors:** Masaya Okura, Souichi Yanamoto, Masahiro Umeda, Mitsunobu Otsuru, Yoshihide Ota, Hiroshi Kurita, Takahiro Kamata, Tadaaki Kirita, Nobuhiro Yamakawa, Tetsuro Yamashita, Michihiro Ueda, Takahide Komori, Takumi Hasegawa, Tomonao Aikawa, Japan Oral Oncology Group

**Affiliations:** ^1^ The First Department of Oral and Maxillofacial SurgeryGraduate School of DentistryOsaka UniversityOsakaJapan; ^2^ Department of Clinical Oral OncologyNagasaki University Graduate School of Biomedical SciencesNagasakiJapan; ^3^ Division of SurgeryDepartment of Oral and Maxillofacial SurgeryTokai University School of MedicineIseharaJapan; ^4^ Department of Dentistry and Oral SurgeryShinshu University School of MedicineMatsumotoJapan; ^5^ Department of Oral and Maxillofacial SurgerySchool of MedicineNara Medical UniversityKashiharaJapan; ^6^ Department of Oral and Maxillofacial SurgeryKeiyukai Sapporo HospitalSapporoJapan; ^7^ Department of Oral and Maxillofacial SurgeryKobe University Graduate School of MedicineKobeJapan

**Keywords:** Bone invasion, head and neck cancer, oral cancer, overall survival, prognosis, squamous cell carcinoma, TNM staging

## Abstract

A multi‐institutional study was undertaken to determine whether mandibular canal (MC) invasion and mandibular medullary bone invasion are independent factors in lower gingival squamous cell carcinoma (SCC). A total of 345 patients with lower gingival SCC were retrospectively reviewed. Mandibular bone invasion was categorized into three types; no bone invasion; invasion through cortical bone (medullary); and MC invasion. The overall survival rate and factors affecting local, regional, and distant failures were assessed by Cox proportional hazards regression analysis and Kaplan–Meier estimates. Bone invasion was present in 201 (58%) patients, of whom 107 (31%) had medullary invasion and 94 (27%) had MC invasion. Using the International Union Against Cancer (UICC) staging system and American Joint Committee on Cancer (AJCC) system, 171 (50%) patients were classified as T4a. When the bone invasion criteria were excluded from the UICC/AJCC system definition, 152 T4a tumors were downstaged and reclassified to T1 in 12 (3%), to T2 in 98 (28%), and to T3 in 42 (12%). In Cox multivariate analysis, MC invasion was an independent predictor of overall survival but medullary bone invasion was not. Medullary bone invasion was an independent variable for distant control. The current T staging system has restricted prognostic utility. The authors recommend a modified T staging system, whereby tumors with MC invasion instead of medullary bone invasion are classified as T4a, and tumors are first classified as T1 to T3 based on size and then upstaged by one T classification in the presence of medullary invasion.

## Introduction

The International Union Against Cancer (UICC) staging system and American Joint Committee on Cancer (AJCC) system for cancers of the oral cavity classify tumors with invasion through cortical bone as T4a. Superficial erosion only of the bone/tooth socket by a gingival primary tumor is not sufficient for classification as T4 1, whereas medullary invasion is classified as T4a. The alveolar gingiva is not a common anatomical site for oral cancer. Gingival cancer arises from the mucosal surface of the oral gingiva. Between the mucosal epithelium and the mandibular bone, there is a thin layer of connective tissue. Because this connective layer has no muscle or muscularis mucosae, invasive tumor cells are immediately able to reach the bone surface. Invasion to the mandibular bone is therefore one of the common features of lower gingival cancer. The incidence rate of T4 tumors was <10% until the 1990s 2, 3, compared with 54–70% in recent studies 4, 5, 6. One possible reason for the increase is the improvement of imaging techniques, including computed tomography and magnetic resonance imaging, to detect bone invasion 7. In 2002, a Japanese epidemiology study on 1804 patients with oral cancer found that 36% of patients with gingival cancer had T4 tumors, which was higher than the rates of tumors at other primary sites in the oral cavity (tongue, 7%; floor of mouth, 18%; buccal mucosa, 20%) 8.

In a previous study, the incidence rate of bone invasion in gingival cancer was high, and bone invasion significantly affected the survival of gingival cancer patients in univariate analyses 4. However, bone invasion was not found to be an independent prognostic factor when confounding variables such as tumor size were taken into consideration, most likely because of the small sample sizes in studies 2, 5. In 2011, Ebrahimi et al. 9. recommended a revision of the T staging system, in which tumors of the oral cavity were first classified as T1–T3 based on size and then upstaged by one T stage in the presence of medullary bone invasion, based on a study of 498 patients with oral squamous cell carcinoma (SCC). This new proposal was followed by Fried et al. 10 in a study of 254 patients with oral SCC. The prevalence of medullary bone invasion was <13% in these studies, because they were conducted in patients with SCC of the oral cavity. A higher incidence of bone invasion was recorded in gingival SCC. Gingival SCC was also associated with elderly patients who were nonsmokers and nondrinkers, and this natural history was different from that of oral SCC at other sites 11. In gingival SCC, there are structural differences, such as the inferior alveolar canal and bone density, between the mandible and the maxilla. In this multi‐institutional retrospective study, we focused on the extent of bone invasion as a prognostic factor in lower gingival SCC and considered the T classification for patients with lower gingival SCC.

## Methods

### Patients

This multicenter study included pooled individual patient data from seven institutions belonging to the Japan Oral Oncology Group 12. Ethics approval was obtained from the institutional review board of each institution. From 2001 to 2012, 426 patients with lower gingival cancer of the oral cavity or mandibular cancer who were treated with curable intent were retrospectively reviewed. All patients had a biopsy‐proven SCC. Patients who had received prior treatment such as neoadjuvant chemotherapy 13 or radiotherapy and those with inadequate information were excluded. A total of 345 patients were eligible for enrollment in this study. Informed consent was obtained from all patients.

### Mandibular bone invasion

All patients underwent pretreatment imaging examinations, including orthopantomography, computed tomography scanning, and/or magnetic resonance imaging. Bone invasion was categorized into three types: no bone invasion, bone invasion was absent or limited to cortical bone; medullary invasion, invasion into cancellous bone was present, but did not extend into the mandibular canal (MC); and MC invasion, invasion extended into the MC. Even partial bone destruction of mandibular canal wall was defined as MC.

### Treatment

Surgery was performed in 334 patients. Concurrent chemotherapy and radiotherapy (CCRT) was performed in 11 patients with advanced tumors that invaded face skin extensively and/or were unfit for surgery. Chemotherapy consisted in CDDP (80–100 mg/m^2^) with or without 5‐fluorouracil (500–1000 mg/m^2^) or oral fluoropyrimidine S‐1 (twice daily 60 mg/m^2^ for 14 days) 14. Radiotherapy was carried out with 40–64 Gy (median: 58 Gy). Marginal mandibulectomy was performed in 163 (47%) patients, segmental mandibulectomy in 140 (41%), and hemimandibulectomy in 31 (9%). Postoperative radiotherapy (median: 52 Gy) was performed in 50 patients with extracapsular nodal spread (ECS), or involved margin. Of these 50 patients, eight underwent adjuvant CCRT with CDDP 12.

### Statistical analysis

Statistical analyses were performed using StatView statistical software (version 5.0; Stata, College Station, TX) and EZR (Easy R) 15. The nonparametric Mann–Whitney *U*‐test was used for continuous variables. Dichotomous variables were compared using the *χ* ^2^ test with Yates correction. The overall survival (OS) rate was calculated by the Kaplan–Meier method, and its statistical significance was analyzed by the log‐rank test. Univariate Cox proportional hazards regression was used to test the associations of bone invasion with local control, regional control, distant control, and OS. Other potential covariates were sex, age, Eastern Cooperative Oncology Group (ECOG) performance status (PS; 0–1 vs. ≥2), T classification, N classification, tumor differentiation, surgical margins (clear vs. close [<5 mm]), number of pathological positive lymph nodes (PLN) and ECS. In the UICC/AJCC staging system, tumor invasion through cortical bone in oral cancer is classified as T4a. Because bone invasion was one of the covariates in this study, bone invasion was excluded from the definition for T4a in T classification. Other invasion factors including invasion into deep extrinsic muscle of tongue or skin of face were classified as T4a. Values of *P *<* *0.05 were considered statistically significant.

## Results

Table 1 shows the patient characteristics. The patients with gingival SCC included 186 (54%) men and 159 (46%) women, with a median age of 70 years (range: 36–93 years). Bone invasion through cortical bone on images was present in 201 (58%) patients, of which 107 (31%) had medullary invasion (nonextension into the MC) and 94 (27%) had MC invasion. According to the TNM classification of the UICC/AJCC staging system (7th edition), 42 (12%) patients had clinical T1 tumors, 88 (26%) had T2 tumors, 14 (4%) had T3 tumors, 171(50%) had T4a tumors, and 30 (9%) had T4b tumors. Because all T4 tumors had medullary bone invasion, the percentage of T4 was >58%. Among the T4a tumors, invasion to skin of cheek was present in 11 (3%) patients and invasion to extrinsic muscle of tongue was present in 8 (2%) patients. The remaining 152 (44%) tumors were classified as T4a by the presence of bone invasion. When the bone invasion criteria were excluded from the definition of T classification, 12 (3%) tumors were reclassified as T1, 98 (28%) as T2, and 42 (12%) as T3. The clinical N classification was N0 in 213 (62%) patients, N1 in 62 (18%), N2 in 69 (20%), and N3 in 1 (<1%).

**Table 1:** Patient clinicopathological characteristics

Variable	No.	%
Age, years		
≤70	173	50.1
>70	172	49.9
Sex		
Male	186	53.9
Female	159	46.1
ECOG performance status (PS)		
PS0–1	260	75.4
PS≥2	22	6.4
Unknown	63	18.3
T classification (UICC/AJCC)		
T1	42	12.1
T2	88	25.5
T3	14	4.1
T4a	171	49.6
T4b	30	8.7
N classification		
N0	213	61.7
N1	62	18.0
N2	69	20.0
N3	1	0.3
Bone invasion		
No bone invasion	144	41.7
Medullary	107	31.0
MC	94	27.2
Tumor differentiation		
Well‐moderately	315	91.3
Poorly	16	4.6
Unknown	14	4.1
Treatment		
Surgery	334	96.8
CCRT	11	3.2
Excision margin		
Clear	184	53.3
Close‐involved	29	8.4
ND	132	38.3
Number of positive nodes (PLN)		
PLN0	232	67.2
PLN1‐2	62	18.0
PN≥3	51	14.8
Extracapsular nodal spread		
Absent	316	91.6
Present	29	8.4
Adjuvant radiotherapy		
Yes	295	85.5
No	50	14.5

ECOG, Eastern cooperative oncology group; MC, mandibular canal; ND, not determined; CCRT, Concurrent chemotherapy and radiotherapy

Medullary invasion and MC invasion were significantly associated with tumor size (*P *<* *0.0001), N classification (*P *<* *0.0001), number of PLN (*P *<* *0.005), and ECS (*P *<* *0.05). These associations were stronger for MC invasion than for medullary invasion. On the contrary, bone invasion was not associated with PS (*P *=* *0.52), tumor differentiation (*P *=* *0.26), and margin status (*P *=* *0.14).

### Survival

**Figure 1:**
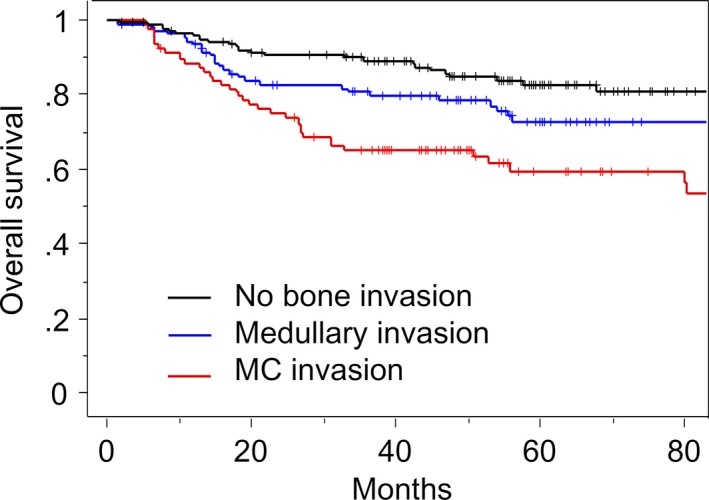
Kaplan–Meier curve of OS according to the type of bone invasion. OS, overall survival.

The median follow‐up was 54 months for all patients, and 61 months for surviving patients. There were 92 deaths. As shown in Figure 1, patients with MC invasion had a significantly lower 5‐year OS of 59%, compared with 73% for patients with medullary invasion and 83% for patients with no bone invasion. OS was significantly lower in patients with MC invasion than those with no bone invasion (*P *<* *0.05).

The results of the Cox univariate proportional hazards analysis are shown in Table 2. MC invasion, age above 70 years, T3–4 (with exclusion of bone invasion criteria), N1, N2–3, poorly differentiated, close margin, number of PLN and ECS were significantly associated with OS. In the multivariate analysis, MC invasion was a significant independent prognostic factor for OS (hazard ratio [HR]: 1.97; 95% confidence interval [CI]: 1.09–3.58). Other independent factors for OS were number of PLN, and ECS.

**Table 2:** Overall survival

Variable	Overall survival			
	Univariate		Multivariate	
	HR (95% CI)	*P*	HR (95% CI)	*P*
Age, >70	1.55 (1.02–2.35)	<0.05	1.53 (0.98–2.39)	0.06
Male	0.95 (0.63–1.42)	0.79	0.98 (0.64–1.51)	0.93
PS≥2	1.43 (0.65–3.11)	0.37	1.21 (0.52–2.39)	0.65
T3‐4^a^	1.64 (1.08–2.49)	<0.05	1.14 (0.71–1.83)	0.60
N classification				
N1	2.02 (1.20–3.39)	<0.01	1.64 (0.90–2.97)	0.11
N2‐3	2.66 (1.65–4.28)	<0.0001	1.04 (0.54–2.04)	0.90
Bone invasion				
Medullary	1.41 (0.83–2.40)	0.21	1.13 (0.61–2.04)	0.68
MC	2.60 (1.59–4.27)	<0.0005	1.97 (1.09–3.58)	<0.05
Poorly differentiation	2.22 (1.07–4.59)	<0.05	1.14 (0.50–2.59)	0.76
Close margin	1.99 (1.05–3.79)	<0.05	1.24 (0.60–2.53)	0.56
No. of PLN				
PLN1‐2	1.79 (1.05–3.07)	<0.05	1.37 (0.73–2.57)	0.33
PLN ≥3	4.19 (2.61–6.68)	<0.0001	3.07 (1.54–6.12)	<0.005
ECS	3.69 (2.11–6.44)	<0.0001	2.34 (1.25–4.36)	<0.01

a After exclusion of bone invasion criteria from the definition of UICC/AJCC system, MC, mandibular canal; No., number; PLN; positive lymph nodes; ECS, extracapsular spread.

**Figure 2:**
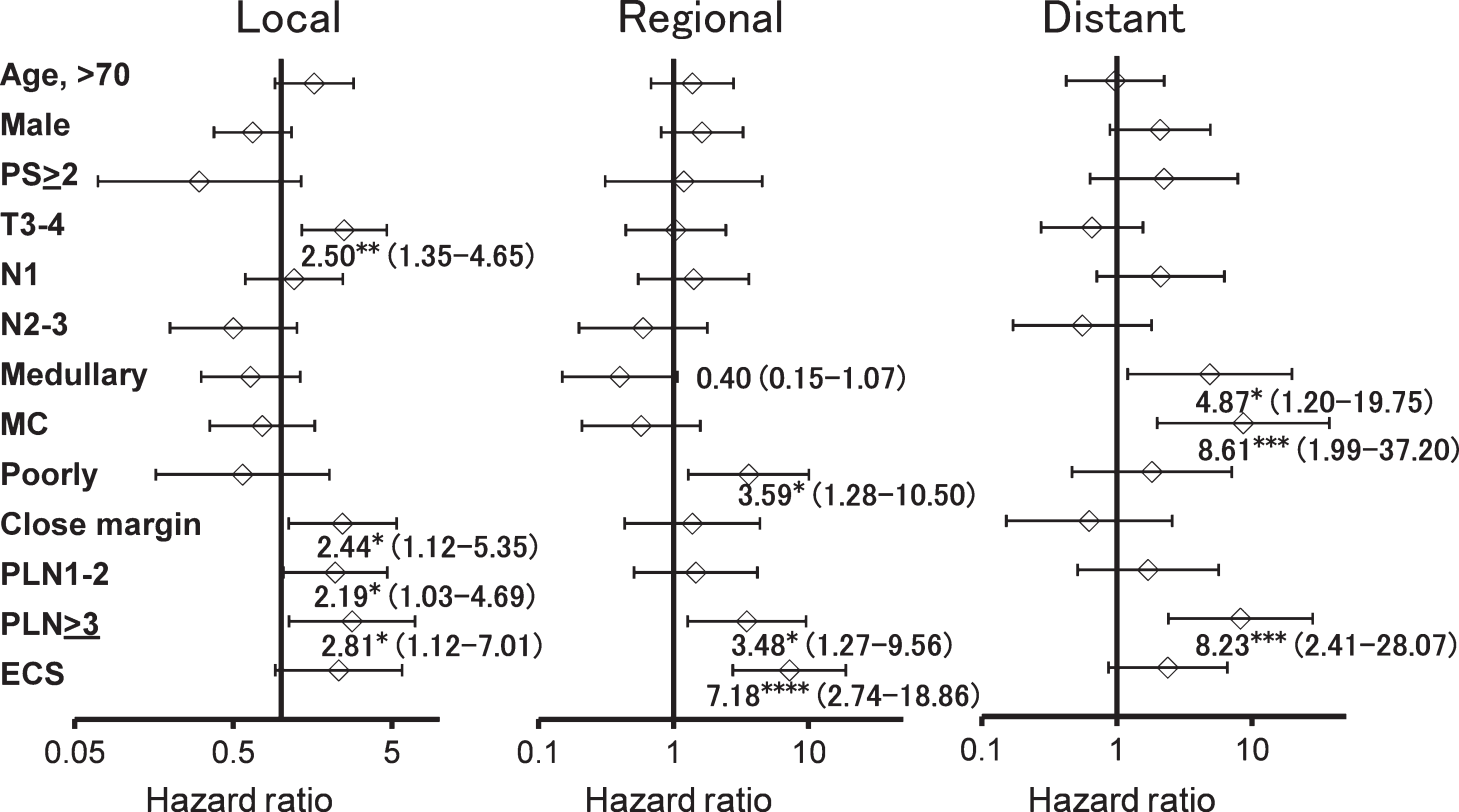
Forest plots showing the multivariate Cox analysis of variables prognostic for local control, regional control, and distant control. The variables included age, sex, PS, T classification, N classification, bone invasion, tumor differentiation, margin status, number of PLN, and ECS. The data are shown with HR (95% CI). *P <* *0.05, **P <* *0.01, ***P <* *0.005, *^***^ *P *<* *0.0001. ECS, extracapsular nodal spread; PLN, positive lymph nodes.

Local failure developed in 56 (16%) patients. Bone invasion was not associated with local control. T3–4, close margin, and number of PLN were significantly associated with local control in the multivariate analysis (Fig. 2). Regional failure developed in 39 (11%) patients. Some patients with N0 neck tumors were not treated by neck dissection, but observed on a wait‐and‐see policy. These patients comprised largely 83 those with no bone invasion, nine with medullary invasion, and nine with MC invasion. Among these patients, delayed lymph node metastasis without local recurrence developed in seven patients with no bone invasion and one patient with MC invasion. No patient with medullary invasion had the delayed lymph node metastasis. Medullary bone invasion was marginally adversely (*P *=* *0.06) associated with regional control, which was likely to be based on patients with delayed lymph node metastasis. Poorly differentiated SCC, PLN≥3, and ECS were significant independent factors for regional control in the multivariate analysis. Distant metastasis developed in 29 (8%) patients. Medullary invasion (HR: 4.87; 95% CI: 1.20–19.75; *P *<* *0.05) and MC invasion (HR: 8.61; 95% CI: 1.99–37.20; *P *<* *0.005) were significantly associated with distant control in the multivariate analyses. PLN ≥3 was an independent factor for distant control.

### T staging

**Figure 3:**
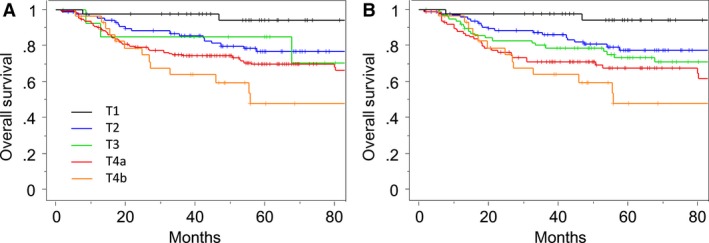
Kaplan–Meier curves of OS according to (A) the current UICC/AJCC T staging system, and (B) the proposed MC T staging system. OS, overall survival.

In the current UICC/AJCC T staging system, 50% of patients had T4a tumors. The Kaplan–Meier analysis for all 345 patients with gingival SCC is shown in Figure 3A. There were overlaps between T2 and T3 tumors for OS. Because MC invasion was an independent prognostic factor for OS, an alternative T staging system was considered, in which T1–T3 tumors were first classified on size and then upstaged by one T stage in the presence of medullary bone invasion according to the proposal of Ebrahimi et al. 9. Tumors with MC invasion were classified as T4a, irrespective of tumor size. The criteria for T4a designation unrelated to bone invasion, including invasion to deep muscle of tongue or skin of face, remained unchanged. According to this MC T staging system, 42 (12%) patients had clinical T1 tumors, 97 (28%) had T2 tumors, 78 (23%) had T3 tumors, 98(28%) had T4a tumors, and 30 (9%) had T4b tumors. Patients with T1 tumors and T4b tumors in the MC staging system were the same as those in the UICC/AJCC staging system. Of the UICC/AJCC T4a tumors, 73 (43%) were downstaged in the MC staging system, comprising nine reclassified as T2 tumors and 64 reclassified as T3 tumors. The survival outcomes according to the revised T staging system are shown in Figure 3B.

## Discussion

This study revealed that MC invasion of lower gingival SCC had a significant impact on survival. MC invasion is one type of invasion through cortical bone, and MC invasion is not included in the definition of the UICC/AJCC staging system. Although the prognosis of oral SCC with medullary bone invasion has been studied, the correlation of MC invasion has not been examined 9, 10. As mandibular bone lies directly below the gingival mucosa, a small gingival SCC can immediately have bone invasion. Tumor cells may readily invade into tooth root tips in dentate patients with periodontal disease. The question therefore arises as to whether small tumors of lower gingival SCC with invasion through cortical bone should be classified as T4a.

In 1993, the medullary bone invasion criteria in gingival SCC were brought up for debate at the Annual Meeting of the Japanese Society of Oral Oncology (JSOO). At that time, the clinical information of 1187 cases with lower gingival SCC was collected and examined retrospectively. In 2004, the committee of the JSOO proposed the Guideline for Clinical and Pathological Studies of Oral Cancer in Japanese 16, which was subsequently published in English in 2012 17. In this guideline, lower gingival cancer with invasion reaching the MC is classified as T4a. Medullary bone invasion is insufficient for T4a. Tumors with invasion into deep muscle of tongue or skin of face are classified as T4a. This staging system for level of MC (LMC) was adopted in 2010, meaning that there are now two staging systems, UICC/AJCC and JSOO LMC, in Japan. Each staging system has advantages and disadvantages. Unfortunately, several cases among the above 1187 patients were treated with the current nonstandard therapies, including preoperative radiotherapy, induction chemotherapy, and radiotherapy. These nonstandard therapies render the evidence for the JSOO staging system insufficient. We therefore conducted this retrospective analysis for the significance of MC invasion on survival.

In the UICC/AJCC staging system, >58% of lower gingival SCC in this study were classified as T4. The Kaplan–Meier method showed overlaps between T2 and T3 in the UICC/AJCC system, and the survival difference between T2 and T4a was very small (Fig. 3A). The 5‐year OS rate was 77% for T2 tumors and 69% for T4a tumors. The rate for T3 tumors was 85%. This inverted outcome is most likely attributed to tumors of ≤4 cm with bone invasion. Among 171 patients with T4a tumors in the UICC/AJCC system, 152 (89%) were classified as T4a based on invasion through cortical bone. When the bone invasion criteria were excluded from the definition of the UICC/AJCC system, a total of 110 tumors of ≤4 cm in size were downstaged from T4a to T1 or T2. This number reflects approximately one‐third of all patients in this study. It is therefore crucial to classify these tumors as either early stage or advanced stage. The corresponding tumors were early stage in size, but had a high risk of distant metastasis because of the presence of bone invasion. Although medullary invasion was not associated with OS in the multivariate analysis, MC invasion was an independent predictor of OS. We therefore believe that tumors with MC invasion should be classified as T4a. Medullary invasion was marginally associated with distant control. For tumors without MC invasion, we recommend the proposal of Ebrahimi et al. 9, in which tumors are first classified as T1–T3 based on size and then upstaged by one T stage in the presence of medullary invasion. This proposed alternative method of T classification is summarized in Table 3. T4a tumors are >4 cm in greatest dimension with medullary bone invasion or invade into the MC, deep (extrinsic) muscle of tongue, or skin of face. Although this MC model is considered to provide better stratification for SCC of the oral cavity, it needs to be validated by further studies.

**Table 3:** Proposed mandibular canal staging system

Primary	Definition
Tumor (T)	
Tx	Primary tumor cannot be assessed
T0	No evidence of primary tumor
Tis	Carcinoma in situ
T1	Tumor 2 cm or less in greatest dimension and no medullary bone invasion
T2	Tumor more than 2 cm but not more than 4 cm in greatest dimension and no bone invasion or tumor 2 cm or less in greatest dimension with medullary bone invasion
T3	Tumor more than 4 cm in greatest dimension and no medullary bone invasion or tumor more than 2 cm but not more than 4 cm with medullary bone invasion
T4a	Tumor more than 4 cm in greatest dimension with medullary bone invasion or tumor invades into mandibular canal, deep/extrinsic muscle of tongue (genioglossus, hyoglossus, palatoglossus, and styloglossus), maxillary sinus, or skin of face
T4b	Tumor invades masticator space, pterygoid plates, or skull base; or encases internal carotid artery

In this retrospective multi‐institutional analysis, we demonstrated that MC invasion is an independent predictor of survival. A retrospective study can have the most sources of error arising from confounding factors and biases of patients. Although a prospective study may be warranted for validation, there are only a few tumors with MC invasion or medullary invasion in the oral cavity. Among oral SCC, tumors of the lower gum have more frequent opportunities for mandibular bone invasion than tumors of other primary sites in the oral cavity. In this study, the prevalence of bone invasion was 58% and that of MC invasion was 27%. The high prevalence allowed us to examine the important correlation between bone invasion, including MC invasion, and prognosis of gingival SCC.

MC invasion was an independent prognostic factor for survival in gingival SCC and was associated with distant metastasis. Medullary bone invasion was also significantly associated with distant metastasis. This association with distant metastasis was consistent with the result for oral SCC in the study by Ebrahimi et al. 9. The association between bone invasion and distant metastasis may be attributed to hematogenous dissemination of tumors that gain access to the circulation via cancellous bone of the mandible. Patients with bone invasion should be considered for adjuvant systemic therapy 9. Ebrahimi et al. 9. in their study of 498 patients with oral SCC and Fried et al. 10. in a study of 254 patients observed that medullary bone invasion adversely reaffected outcomes, and recommended that tumors should first be classified as T1–T3 based on size and then upstaged by one T stage in the presence of medullary invasion. Although these studies did not mention MC invasion by SCC of the oral cavity, the proposed MC method might be effective for oral SCC as well as gingival SCC.

Bone invasion was not associated with local control, while large tumor size, closed margin, and number of PLN had a significantly higher risk of local recurrence. Patients with bone invasion had an inversely lower risk of regional failure, which was likely to be caused by delayed neck lymph metastasis in patients whose neck tumors were not treated by elective neck dissection, but observed on a wait‐and‐see policy 18. Marginal mandibulectomy is often difficult to adapt for tumors with bone invasion, but is an oncologically safe procedure for some tumors with no bone invasion. In this study, 70% of patients with no bone invasion underwent intraoral marginal mandibulectomy without elective neck dissection. These patients may harbor occult metastases. The occult metastatic rate was no more than 9% in this study, meaning that a wait‐and‐see policy is warranted 18. Poorly differentiated SCC, PLN≥3, and ECS had worse regional control. This study population had only 16 (4.8%) cases of poorly differentiated SCC (Table 1), which might warrant further investigation for validation.

PLN ≥3 was an independent prognostic factor for OS, and was significantly associated with local, regional, and distant control in multivariate analysis in this study. The presence of pathological positive nodes is the most important prognostic factor for oral SCC 9, 12, 13. Tumors with ECS had an extremely worse prognosis 19, 20. The prevalence of ECS in this study was 8.4%, being relatively low compared with other primary tumors of the oral cavity 21. In addition, the prevalence of closed margin was similarly 8.4%. Those patients with ECS and/or closed margin were candidates for adjuvant RT. However, patients with pT3 or pT4 were not the candidate in this study, because medullary bone invasion was questionable criteria for T4a classification. ECS was an independent variable for regional control, and OS in the Cox multivariate proportional hazards model. MC invasion was also an independent prognostic factor for OS. We therefore recommend the proposed MC staging system for T classification of lower gingival SCC.

## Conclusions

In this retrospective multi‐institutional analysis, we demonstrated that MC invasion is an independent predictor of reduced survival for patients with lower gingival SCC. Medullary bone invasion is not a significant predictor of survival, but is significantly associated with distant metastasis. In the current UICC/AJCC T staging system, the proportion of T4a tumors is high for gingival SCC and the system appears to have restricted prognostic utility. Modifications might be warranted.

## Conflict of Interest

We have no conflict of interest to declare.
